# Types of pain and their psychosocial impact in women with rheumatoid arthritis

**DOI:** 10.1186/s40695-019-0047-4

**Published:** 2019-08-09

**Authors:** Maria Gabriela Chancay, Shirin Nouri Guendsechadze, Irene Blanco

**Affiliations:** 1 Department of Rheumatology, Albert Einstein College of Medicine, 1300 Morris Park Ave, Forchh 701N, Bronx, NY 10461 USA; 2 Department of Medicine, Jacobi Medical Center, 1400 Pelham Pkwy S, Bronx, NY 10461 USA

**Keywords:** Rheumatoid arthritis, Non-inflammatory pain, Women, Middle age

## Abstract

Rheumatoid arthritis (RA) is a systemic inflammatory autoimmune disease predominantly affecting middle-aged women. Very commonly, pain is a manifestation of active disease and because untreated RA can result in joint deformities, the current evaluation of pain has largely focused on inflammation. In addition, treatment has centered on the premise of reducing disease activity with the hopes of halting worsening damage, preventing future deformities, and ultimately providing pain relief for the patient. Yet research shows that all patients with RA, but women in particular, often suffer from increased mechanical pain and fibromyalgia, as well as anxiety, depression, sleep disturbances, sexual dysfunction, and disability, which add to the burden of the illness. Determining and addressing alternative pain triggers as well as understanding the psychosocial burden of RA is key in treating patients, especially in those who may not improve with traditional pharmacotherapy.

## Introduction

Rheumatoid arthritis (RA) is a chronic, systemic, inflammatory disease, classically affecting the small joints of the hands and feet [1]. In industrialized countries, RA affects 0.5–1.0% of adults and there are approximately 5–50 new cases per 100,000 adults per year [2]. Like most autoimmune diseases, RA primarily affects women with a female to male ratio of 3:1 [2, 3]. Among men younger than 45 years of age, RA is rare while it occurs four times more often in women who are less than 50 years old [3–5]. With age, the incidence of RA among men increases but so does the incidence of RA among women, which peaks around menopause [3–5]. While the disease can occur in either sex at any age, it is predominately a disease of middle-aged women. Like men with RA, these women are at increased risk for cardiovascular events and are at increased risk for total mortality [6].

The discrepancy in the incidence and prevalence of RA among the sexes suggests that there are factors associated with the female sex that play a role in the development and progression of RA, with the literature focusing primarily on a hormonal component. Studies on hormonal differences, however, have yielded conflicting results. Pregnancy and breastfeeding, for example, have been associated with a decreased risk of developing RA [4, 7, 8]. Concomitantly, the post-partum and post-menopausal periods, and in particular, early-onset menopause (less than 44 years old) have been associated with an increased risk [4, 7, 9]. This would suggest a protective role of estrogen in RA, yet this is inconsistent with the fact that the disease is more common in women versus men and that studies of hormone replacement therapy and oral contraceptives have shown inconsistent results [8, 10, 11].

A critical issue in the management of RA is recognition that not all pain in RA is due to active disease. Patients with RA also experience non-inflammatory pain which includes mechanical pain (like osteoarthritis), neuropathic pain, fibromyalgia, side effects of treatment, as well as the psychosocial sequela from the disease like depression, anxiety, sleep disturbances, sexual dysfunction, and disability [1, 6, 12, 13]. Given the available literature, this review will focus on how women are affected by these non-inflammatory pain generators. Secondly, it seeks to encourage clinicians and researchers to take a broader approach to pain when working with patients with RA.

Currently the focus in RA treatment is to decrease inflammation and achieve remission in an effort to prevent deformities and erosions. While this should certainly be a goal in treatment, a reduction of inflammatory pain may not be enough to address the myriad of ways RA drastically affects patient’s lives. Failing to recognize that inflammation is not the only cause of pain in RA can lead to unnecessary changes in therapy and a lack of attention to non-inflammatory targets of pain management and psychosocial support [14].

## Pathophysiology of rheumatoid arthritis

### A. Mechanism

The pathophysiology of RA is complex, multifactorial, and is not yet fully understood. The current hypothesis is that in genetically susceptible individuals, environmental factors can trigger an aberrant activation of the immune system, including excess production of tumor necrosis factor-alpha (TNF-alpha) and interleukin-1 (IL-1), leading to the clinical onset of disease [4, 12]. In RA, approximately two-thirds of the risk of developing the condition is attributed to genetic factors—mainly the HLA-DRB1 alleles which encode a five-amino acid sequence motif known as the shared epitope (SE) [15, 16]. It remains unclear how exactly the shared epitope interacts with environmental factors and leads to the RA inflammatory cascade, although viral infections, commensal bacteria, and cigarette smoke among others have been implicated [15]. It has been suggested that in patients with the SE, cigarette smoke may activate protein citrullination in the lungs, which can become antigenic and form anti-citrullinated protein antibodies (ACPA). These proteins contribute to the dysregulation of the immune system [15].

### B. Clinical presentation

Although a multi-systemic problem, RA classically presents as a chronic inflammatory disease of synovial joints. It typically manifests as polyarticular pain with a symmetric distribution mainly affecting the hands and the feet. In the hands, it has a preference for the wrists, as well as the metacarpophalangeal and proximal interphalangeal joints. These joints are not only painful but also often swollen and warm. Morning stiffness lasting for more than 30 min is typically present. Rheumatoid factor (RF) and ACPA are often seen in addition to elevated acute phase reactants, i.e. sedimentation rate and c-reactive protein. About 50 to 80% of RA patients have RF, ACPA, or both and the presence of these antibodies are associated with worsened prognosis [2]. Untreated, RA can lead to joint deformities with irreversible bone and cartilage destruction leading to disability and a decreased quality of life [12]. Extra-articular manifestations can occur, especially in those that are untreated, examples are: rheumatoid nodules, interstitial lung disease, vasculitis, mononeuritis multiplex, and scleritis [2]. RA can also be seen in association with other conditions such as Felty’s syndrome(RA, splenomegaly, and neutropenia) and Caplan’s syndrome(RA and pneumoconiosis). In some cases, RA can exist in conjunction with other autoimmune diseases including Sjögren’s syndrome and systemic lupus erythematosus [17].

Despite the extra-articular manifestations that RA can have, it most commonly presents with joint pain and morning stiffness. RA activity is evaluated by using composite measures like the clinical disease activity index (CDAI) or the simplified disease activity index (SDAI), which are composed of the number of swollen joints, tender joints, the patient global assessment, and the clinician global assessment, with the SDAI also including the c-reactive protein. These disease indices include both objective and subjective measures (the number of tender joints and patient global assessment) and traditionally have assumed that all factors are primarily driven by the inflammatory component of RA.

Several studies have shown that elevated composite measures that include patient-reported components can also reflect the presence of non-inflammatory pain [14]. A cross-sectional study in Italy of 292 patients with RA showed that patients with comorbid fibromyalgia compared to patients with RA only, had higher tender joint counts and DAS 28 score (another disease activity score similar to CDAI or SDAI) despite having similar disease activity as measured by rates of erythrocyte sedimentation rate and swollen joint counts [18]. In Sweden, a separate cross-sectional study found that one-third of 102 women with early RA also met criteria for widespread pain. The women with widespread pain reported a higher number of tender joints, greater pain intensity, worse global health, activity limitation, fatigue, depression, and anxiety. This is despite inflammation being similar between the groups [13]. This and other studies looking at sleep, neuropathic pain, and emotional health suggest that there is a relationship between RA and non-inflammatory pain generators. The latter seems to contribute to the intensity of pain and also add to psychosocial stress, which in turn can also act as a non-inflammatory pain trigger and affect the way RA is experienced [2, 13, 14, 19–22]. In one study, for example, women with RA and widespread pain were found to have reduced hand-grip force and muscle endurance, compared to women with RA only, despite the fact that disease activity was similar. The study then suggests that these women are at a higher risk for activity limitation in the future as a result of their decreased force and endurance [13].

## Non-inflammatory pain generators in rheumatoid arthritis

### A. Mechanical pain

While active RA can cause inflammatory pain, discomfort can continue even after inflammation has subsided. Bone and cartilage destruction can lead to the development of secondary osteoarthritis resulting in mechanical pain despite disease remission or low disease activity [12, 23]. One would suspect that the introduction of biologics leading to better disease control would decrease total joint replacement however results have varied depending on joint involvement (especially in terms of total knee replacement) and across countries [23–26]. Some studies have shown a decrease in the incidence of total knee replacement in recent cohorts, while others have shown a stable or even slightly higher rate [23, 24, 27]. Reasons to explain the stable or higher knee arthroplasty rates among contemporary cohorts includes the presence of osteoarthritis despite remission of RA as well as patients with RA now being able to achieve similar pain and function outcomes, when compared to patients with primary osteoarthritis, thanks in part to their ability to participate in physical therapy due to better disease control [28].

Unfortunately, both degenerative changes and active inflammation can necessitate joint replacement and often it is difficult to determine whether joint damage is the result of subclinical or undetected activity versus osteoarthritis. In a study looking at two United Kingdom inception cohorts (1986–1999, 2002–2012) with early RA and on treatment, one of the main findings was that a high proportion of patients required surgical procedures (29%) with major surgeries (knees, hips) accounting for 35% of all procedures and intermediate surgeries (wrists, hands, hindfoot/forefoot) accounting for 24% [23]. Interestingly, while the 10-year cumulative incidence of major interventions remained stable, there was a significant decline in intermediate surgeries. The study also showed that over time there was a trend away from monotherapy and towards combination disease-modifying antirheumatic drugs (DMARDs) therapy including the addition of biologics. One of the questions postulated by the study was whether the unchanged incidence of major intervention with time could be explained by several factors including the presence of osteoarthritis despite better control of RA [23]. Several studies have also shown that women are more likely to undergo joint replacement than men although the exact reason why is unclear [24, 25, 29].

### B. Pain as sequela of treatment

DMARDs are the mainstay of treatment and the way clinicians try to halt the progression of RA, however they often take time to work and so for more immediate relief, glucocorticoids (GCs) are used. GCs work by quickly decreasing inflammation and synovitis. Long-term, they can also decrease joint damage but are associated with numerous side effects including infection, diabetes, hypertension, adrenal insufficiency, and osteoporosis [2, 30]. They can also indirectly contribute to non-inflammatory pain by causing changes in body habitus and mood disturbances [31]. They place patients at higher risk for avascular necrosis, fractures, and steroid myopathy [30]. Middle-aged women with RA are at higher risk for fractures given their increased risk for osteoporosis [32]. Due to their adverse side effects, the use of GCs should be limited to the initiation of therapy and during episodes of disease exacerbations, and should be used sparingly as adjunct therapy while DMARDs take effect [33].

Similarly to GCs, all medications for RA can contribute to non-inflammatory discomfort if their side effects outweigh their benefits. DMARDs, particularly the biologics, can also increase the risk of infections [34]. Non-steroidal anti-inflammatory drugs (NSAIDs) can cause gastrointestinal problems including dyspepsia and bleeding. Medications such as duloxetine, gabapentin, and the tricyclic antidepressants that are used for concurrent generalized pain/fibromyalgia can lead to sedation, weight gain, and sexual dysfunction [35]. Opioids should be avoided as their side effect profile not only includes sedation and GI discomfort but can also lead to dependence and opioid-induced hyperalgesia [12, 36, 37].

### C. Pain due to fibromyalgia

Fibromyalgia (FM) is one of the most common “rheumatic” disorders after osteoarthritis with a suspected prevalence of 2 to 8% of the population, where it affects women twice as commonly as men [35]. About 12 to 48% of RA patients have concurrent FM, which can be seen within the first year of diagnosis [38]. Although the exact etiology of FM is unknown, it is considered to be a centralized pain disorder. Patients with FM often report widespread, chronic pain and have a lower threshold for painful stimuli [35]. As previously mentioned, current composite measures of RA disease activity can be affected by the presence of concomitant FM. In fact, several studies show that not all pain in RA is associated with objective measures of inflammation, and this is true particularly in women, although the exact etiology behind this has not been elucidated [6, 13, 14, 18]. Women with RA and FM tend to report higher tender joint counts and worse global health status compared to those without FM [18]. As the current focus of treatment is on reducing inflammatory pain, the immediate reaction is to attribute any discomfort to higher disease activity [6]. A prospective cohort study of patients with RA and FM showed that these patients were treated with more leflunomide and prednisone than RA patients without FM [39]. It is especially important to differentiate FM pain from inflammatory pain as it can confound disease activity indices leading to unnecessary changes in DMARDs and delaying appropriate therapy.

### D. Pain due to sleep disorders

Both FM and RA patients often have issues with sleep and fatigue at higher rates than the general population [20, 35, 38]. RA on its own has been associated with poor sleep quality in about 50 to 70% of patients, in the form of sleep fragmentation, non-restorative sleep, obstructive sleep apnea (OSA), and restless leg syndrome [40–42]. Current literature suggests a bidirectional relationship between sleep and pain where pain can cause sleep disturbances and lack of sleep can result in worsening perception of musculoskeletal pain [20, 43]. Both non-optimal sleep and worsening pain have been associated with a decrease in quality of life [42]. In a self-administered questionnaire study involving patients with RA, sleep fragmentation was the most common sleep abnormality reported. The number one reason cited for the nighttime awakening was the “need to use the washroom” followed by pain experienced at night [40]. In this population, poorly controlled pain directly affected patients’ quality of sleep. It would have been interesting to see if the number one reason for nighttime awakening was a result of medication side effects. Medications like steroids and certain anti-depressants can cause insomnia, dry mouth, and other side effects that may contribute to frequent bathroom visits.

While the discomforts associated with RA can cause sleep disturbances, poor sleep has also been shown to increase joint pain independent of anxiety and depression [14, 20]. For example, patients with OSA have greater disease activity as represented by DAS28 and higher levels of c-reactive protein [44]. In an experimental model of partial night sleep deprivation comparing patients with RA and control subjects, patients with RA reported worsening pain and an increase in the number of affected joints after sleep loss. Additionally, they had worsening fatigue, anxiety, and depression, which have also been associated with worsened, non-optimal sleep [20]. Given the close relationship between pain, sleep, and mood, it is important to ensure that these non-inflammatory causes of pain are appropriately addressed in order to help with disease control and improve quality of life.

## The psychosocial impact of non-inflammatory pain in women with rheumatoid arthritis

### A. Mood disturbances and anxiety in RA

Unfortunately, patients with chronic illnesses are at higher risk of developing mood disorders and anxiety [45]. RA, like hypertension and diabetes, appears to elevate this risk with the incidence and prevalence of depression, anxiety, and bipolar disorder, being higher, especially among women [46]. As with sleep, there seems to be a bidirectional association between RA and depression. RA increases the risk of depression and depression can increase the pain associated with RA by increasing pain sensitivity [47, 48]. Post-traumatic stress disorder (PTSD) has also been shown to increase the number of tender joints and exacerbate the pain felt by patients with RA [49, 50]. It is important then to recognize the emotional burden that RA places on its patients as it can interfere with function, reduce medical adherence, contribute to maladaptive health behaviors, and create risk for greater disease activity and medical co-morbidities [51].

### B. Sexual health

Sexual dysfunction is a highly prevalent problem in patients with RA [52–54]. It is estimated that more than half of RA patients will experience some degree of sexual dysfunction over the course of their lifetimes [52]. Causes are thought to be multifactorial including age, disease duration, sequelae of disease and psychosocial morbidities such as depression, low self-esteem, and decreased body image [53–56]. Limitations with the hips and knees can make it difficult for women with RA to engage in sexual activities and can have a detrimental effect in their quality of life and relationships [54, 56, 57]. After controlling for disease activity and pain intensity, physical performance has been directly related to sexual activity and satisfaction [54].

In addition, patients with RA, especially those with aggressive disease, often experience changes in their physical appearance and function [58]. For example, women with higher degree of morning stiffness have been shown to be more concerned over their body image and have reported more sexual dissatisfaction [59]. Women experiencing weight gain from GCs also report having a poorer perception of their bodies [56]. This can lead to low sexual desire, fear of pain, and a fear of an inability to engage in a satisfying sexual relationship [52].

### C. Disability

In many individuals, RA can cause difficulties with daily activities. Nearly 85% of patients have trouble doing household chores, and often women require more assistance than men [60]. The majority of patients (close to 60%) also report reducing or completely giving up a leisure-time activity because of their RA [60]. Loss of leisure-time activities has been associated with more fatigue and pain, as well as less self-efficacy in coping with RA [61]. Patients report that their disease makes them less independent and interferes with their activities like working, participating in hobbies, and having support, thereby reducing their quality of life [1, 60, 62]. Disability also causes a loss of income to the individual and to society at large. It is estimated that there is a total annual societal loss of approximately $40 billion dollars when taking intangible and indirect costs into account [63].

Like depression and sleep disorders, disability causes psychosocial stress affecting the perception of pain. In the BARFOT study, two cohorts of patients with early RA were followed for 8 years with a focus on functional disability and pain [64]. Despite the second cohort being exposed to more aggressive medical treatment and having lower disease activity, pain and functional limitation of both groups were the same, suggesting once again that other factors, apart from disease activity, affect the way pain is interpreted.

The Health Assessment Questionnaire (HAQ) is typically employed to assess physical function. It is a validated questionnaire focusing on daily activities that include eating, dressing, and hygiene among others. A worsening HAQ score has been seen with longer disease duration and is considered to be a predictor of future disability and mortality [5, 65, 66]. Among women, those who are post-menopausal seem to have increased disability as assessed by HAQ. This difference does not appear to be due to radiographic joint destruction suggesting that the functional decline seen in this population is not primarily due to damage and may be related to changes surrounding menopause [5]. Unfortunately, worsening HAQ scores have also been associated with a decrease in quality of life and negatively impacted relationships [67]. The loss of function and disability experienced by patients with RA can worsen depression and sleep disorders, and contributes to social isolation, and a worsened perception of pain [19].

## Treatment

Early in the course of the disease, there can be minimal pain as well as minimal damage to the bone and cartilage, however as the disease progresses it can lead to significant deformities. This is why the focus of treatment has remained on decreasing inflammatory pain. DMARDs have been the cornerstone of therapy as they not only help to alleviate inflammatory pain but prevent further joint damage while improving physical function [16]. DMARDs, as listed in Table 1, are divided into conventional and targeted synthetics, and biologic agents. Older medications, like methotrexate, hydroxychloroquine, leflunomide, azathioprine, cyclosporine, and sulfasalazine are conventional synthetic DMARDs [16]. The targeted synthetic DMARDs are new agents and include tofacitinib and baricitinib, and are known to work by interfering with cell signaling. Biologic agents include the injectables and the infusions such as the TNF-inhibitors (e.g. etanercept, adalimumab, infliximab), rituximab, abatacept, the interleukin-6 receptor antagonists tocilizumab and sarilumab, and the interleukin-1 receptor antagonist anakinra.

**Table 1:** Currently Approved Disease-Modifying Antirheumatic Drugs

1. Synthetic DMARDs	
Conventional Synthetic DMARDs	Methotrexate, Sulfasalazine, Leflunomide, Hydroxychloroquine
Targeted Synthetic DMARDs	Tofacitinib, Baricitinib
2. Biologic DMARDs	
TNF-inhibitors	Etanercept, Adalimumab, Infliximab, Golimumab, Certolizumab
Anti-B-cell	Rituximab
Anti-T-cell	Abatacept
Anti-IL-6 receptor	Tocilizumab, Sarilumab

Opioids and NSAIDs are not sufficient in stopping disease progression despite the fact that they can help control pain and as such are not stand-alone therapies for RA. Similarly, steroids, while they can quickly decrease inflammatory pain, should only be used as an adjunct to DMARD therapy. Both the American College of Rheumatology and the European Union League Against Rheumatism recommend a “treat to target” approach, with a goal of decreasing 50% of RA activity by 3 months and reaching remission by 6 months of diagnosis. If these targets are not met, a different therapy should be considered [68]. It is this goal of decreasing disease activity that can drive clinicians to switch medications or step up therapy if pain persists; and why it is so essential that the correct type of pain is identified.

Treatment of inflammatory pain remains the focus of most clinicians, yet given the different pain generators in RA and their relationship to one another, it is essential to recognize and embrace the modalities used to treat non-inflammatory pain. Physical and occupational therapy are already widely used and can help decrease both inflammatory and non-inflammatory pain through muscle strengthening and stabilization of the joints.

Patients with RA can often experience “rheumatoid cachexia” which occurs as a rapid loss of muscle mass due to protein degradation from excess of proinflammatory cytokines [69]. Body weight remains stable but the loss in muscle mass contributes to weakness, fatigue, and decreased movement. These changes place the patient at risk for functional disability and worsen inflammatory pain by having patients change their natural posture and the way they move in order to compensate for the painful joint. High intensity resistance exercise can help reverse cachexia by increasing muscle mass and strength while helping to stabilize joints and preventing contractures [69, 70]. Physical activity may also promote health of the joint by increasing joint lubrication during exercise [69].

Physical and occupational therapy can help with non-inflammatory pain by decreasing the risk of disability and increasing independence [71]. Physical activity in general, even unsupervised, has been associated with a positive influence in quality of life [72]. Occupational therapy can help patients improve their ability to perform a functional task, allowing them to achieve independence and autonomy. Preservation of autonomy is essential in patients with RA as it decreases anxiety and preserves psychological well-being [22]. Other modalities shown to improve quality of life and promote functional benefits include electrotherapy and mud therapy. Aquatic therapy may have positive effects in patients with RA who are unable to engage in traditional physical therapy [73–75].

Like other forms of chronic pain, the pain in RA is multifactorial and treatment options can vary. Cognitive behavioral therapy (CBT) has been studied in patients with chronic pain conditions, chronic low back pain, osteoarthritis, fibromyalgia, as well as in different psychiatric disorders including depression, PTSD, and generalized anxiety disorder [76]. It is also first-line treatment for chronic insomnia [77]. CBT focuses on identifying and changing behaviors and thoughts that are maladaptive while helping patients develop coping strategies. It may help to ameliorate pain and disability by changing attitudes towards the illness. In a study looking at patients with recent onset (< 2 years) of seropositive RA, those that receive CBT had a reduction in depressive symptoms and c-reactive protein as well as an improvement in joint involvement compared to those who did not receive CBT [78]. Other studies have shown that CBT can reduce disability, depression/anxiety, and pain-related fear but do not decrease pain intensity [79]. Interestingly, CBT may also change brain function and neuronal connection as magnetic resonance imaging done after treatment with CBT show increase activation in the prefrontal cortex, an area of the brain that is involved in regulating emotions to painful stimuli [76].

In recent years, complementary/alternative medicine (CAM) such as acupuncture has been used in the treatment of chronic conditions like RA [80]. Acupuncture may provide short term analgesia by creating up-regulation of endogenous opioids and serotonin [81]. One study showed that it can help improve quality of life by decreasing the number of tender joints although this effect is short-lived [80].

Fibromyalgia should be addressed if present. Its treatment includes both pharmacologic and non-pharmacological approaches [82]. The non-pharmacological treatments include CBT, aerobic exercises, tai-chi, yoga, hydrotherapy and acupuncture [82, 83]. Most recently, tai-chi showed similar or greater improvement in symptoms than aerobic exercises which is the current most commonly prescribed non-pharmacological treatment modality for FM [84]. Combining various modalities has also been shown to be effective in decreasing pain, fatigue, and depression, and can also improve quality of life although effects are not long-lasting [83].

Pharmaceutical therapies for FM include amitriptyline (a tricyclic antidepressant) and anticonvulsants like gabapentin and pregabalin [85, 86]. Treatment options also include the serotonin-noradrenaline reuptake inhibitors (SNRIs): duloxetine and milnacipran. In a subgroup analysis of patients with RA with well-controlled inflammation and widespread pain, milnacipran was found to decrease pain after 6 weeks of medication use [87]. As fibromyalgia and depression can often co-exist, duloxetine (which has a stronger effect on serotonin than does milnacipran) is an ideal choice for these patients [85, 86]. In individuals with RA and stand-alone anxiety and/or depression, the clinician may also wish to consider using selective serotonin reuptake inhibitors (SSRIs) like fluoxetine and encourage physical activity. Other pharmaceutical modalities for fibromyalgia include cyclobenzaprine [85, 86]. The use of opioids is discouraged because of the lack of efficacy in FM patients in addition to the high risk of side effects and addiction [85].

Patients with RA who are experiencing sexual dysfunction due to difficulties with sexual intercourse should be encouraged to have a open discussion with their partners as communication can help everyone feel more comfortable. Patients can also find different positions and/or explore alternative ways of expressing sexuality. Patients can also try analgesics, heat, and muscle relaxants before engaging in sexual activity. Vaginal lubrication and estrogen cream can be used for dyspareunia. Those experiencing decreased libido should be ruled out for medication side effects and depression. Anti-depressants, medications such as sildenafil, and sex therapy can be pursued as needed [52, 88].

Sleep disorders should also be addressed as they can negatively impact well-being and function [42]. At times, patients are not even aware that they have poor sleep. In a study looking at thirty female patients with RA, fourteen met the criteria for diagnosis of OSA despite six of them reporting not having any sleep problems [44]. Patients with RA and fatigue and/or poor sleep, benefit from an evaluation for restless leg syndrome and OSA. After diagnosis, continuous positive airway pressure or pharmacological therapy for restless leg syndrome can be pursued. Other types of sleep issues include insomnia and non-restorative sleep. Medication side effects should be ruled out and sleep hygiene should be encouraged. CBT can be used if insomnia persists, and in fact is first line therapy [89]. Studies have shown that 70 to 80% of patients benefit from CBT and approximately 40% achieve remission from insomnia [90]. While it is common to use antidepressants to help improve sleep, there are not enough quality studies to determine whether the effect is significant although they may work better if poor sleep is a result of depression [91]. Hypnotic drugs, like benzodiazepines, should be avoided as they can induce tolerance and dependence and in the elderly population can cause falls [91, 92].

## Take away points and conclusion

RA is described as a chronic, systemic, inflammatory disease that affects mainly the joints of the hands and the feet. Typically, active disease presents with pain and when left untreated can cause joint deformities and disability. In this context pain is often used as a proxy for active disease. Traditionally, the evaluation of pain in RA has focused on inflammation yet patients with RA also experience non-inflammatory pain such as mechanical pain (like osteoarthritis), neuropathic pain, fibromyalgia, side effects of treatment, as well as the psychosocial sequela from the disease like depression, anxiety, sleep disturbances, sexual dysfunction, and disability [1, 6, 12, 13]. Often there is also a reciprocal relationship between disease and the non-inflammatory pain generator. Mood and sleep in particular have a bidirectional relationship with active disease [20, 47].

Given the current approach in treating RA, a significant amount of women’s pain is not being adequately addressed. Moreover, when asked to choose an area of health that they wished would improve, close to 70% of women choose pain even above walking, mobility, and hand and finger function [93]. This suggests that more attention needs to be paid to patients' preferences. Clinicians may be happy with the lack of synovitis while patients suffer due to depression or fibromyalgia. When left untreated, non-inflammatory pain, like joint deformities can have tremendous impact on patients’ quality of life and mobility [60, 61]. Ultimately, in order to recognize the various ways in which RA can cause pain, providers need to broaden their sense of what RA is and how patients experience the disease. Only by reimagining RA, can non-inflammatory causes of pain be recognized and be treated accordingly.

## Data Availability

Not applicable

## Abbreviations

ACPA: Anti-citrullinated peptide antibody
CAM: Complementary/alternative medicine
CBT: Cognitive behavioral therapy
CDAI: Clinical disease activity index
DMARDs: Disease-modifying antirheumatic drugs
FM: Fibromyalgia
GCs: Glucocorticoids
HAQ: Health Assessment Questionnaire
IL-1: Interleukin-1
NSAIDs: Non-steroidal anti-inflammatory drugs
OSA: Obstructive sleep apnea
PTSD: Post traumatic stress disorder
RA: Rheumatoid arthritis
RF: Rheumatoid factor
SDAI: Simplified disease activity index
SE: Shared epitope
SNRIs: Serotonin-noradrenaline reuptake inhibitors
SSRIs: Selective serotonin reuptake inhibitors
TNF-alpha: Tumor necrosis factor-alpha
